# A rare mosaic 22q11.2 microdeletion identified in a Chinese family with recurrent fetal conotruncal defects

**DOI:** 10.1002/mgg3.847

**Published:** 2019-07-11

**Authors:** Weicheng Chen, Xiaodi Li, Liqun Sun, Wei Sheng, Guoying Huang

**Affiliations:** ^1^ Cardiovascular Center Children's Hospital of Fudan University Shanghai China; ^2^ Department of Ultrasound International Peace Maternity and Children Health Hospital Shanghai China; ^3^ Shanghai Key Laboratory of Birth Defects Shanghai China

**Keywords:** 22q11.2 microdeletion syndrome, chromosomal microarray analysis, fluorescence in situ hybridisation, mosaic fetal conotruncal defects

## Abstract

**Background:**

22q11 deletion syndrome (22qDS) is caused by deletion of chromosome region 22q11.2. However, mosaic cases with 22q11.2 deletion syndrome (22q11.2DS) are rarely reported.

**Methods:**

Chromosomal microarray analysis (CMA) and fluorescence in situ hybridization fluorescence in situ hybridization (FISH) were performed to analyze the copy number alterations. Clinical examinations related to 22q11.2DS were performed on the carrier in this family.

**Results:**

A healthy female in a Chinese family with a history of two pregnancies with conotruncal defects, one with pulmonary atresia (PA) and another with Tetralogy of Fallot (TOF) was recruited in this study. CMA revealed that the fetus with TOF has a microdeletion on the 22q11.2 locus, and his mother was further confirmed a somatic mosaicism of 22q11.2 microdeletion by interphase FISH. Somatic mosaic 22q11.2 deletion in the mother was validated in the metaphase lymphocytes. Clinical examinations related to 22q11.2DS showed that the mother had hypocalcemia and low percentages of CD4 + T helper cells. The family history of recurrent fetal conotruncal defects and genetic results demonstrated the inherited possibility of maternal germline mosaicism of the 22q11.2 microdeletion.

**Conclusion:**

Our report was the first case in a Chinese family to present that a somatic and suspected gonadal mosaicism of the 22q11.2 microdeletion in female causes recurrent fetal conotruncal defects.

## 1 INTRODUCTION

22q11.2 deletion syndrome (22q11.2DS) refers to a syndrome that results from deletion of the 22q11.2 region, affecting approximately one in every 4,000 to 6,000 newborns and one in every 1,000 unselected fetuses (Kruszka et al., 2017). The phenotype of 22q11.2DS is highly variable and includes the following multiorganism anomalies: cardiovascular anomalies, immunodeficiency, endocrine abnormalities, renal abnormalities, developmental delays, and behavioral and mental disorders (McDonald‐McGinn et al., 2015). Cardiovascular abnormalities are often the initial manifestation of 22q11.2DS, especially conotruncal defects, which include tetralogy of Fallot (TOF), interrupted aortic arch, and truncus arteriosus (Peyvandi et al., 2013).

The de novo deletion of 22q11.2DS was found in 90%–95% of identified patients, which means that there were 5%–10% cases with an inherited deletion (McDonald‐McGinn et al., 2015). Mosaic microdeletion syndrome has been reported as case reports and is considered to be rare, but a study indicated that mosaicism in 22q11.2DS is common with a frequency of 28.2% (Halder, Jain, & Kalsi, 2018). Mosaicism refers to the presence of more than two cell population genotypes in one individual that results from chromosomal nondisjunction, anaphase lagging, and endoreplication (Taylor et al., 2014). Happening prior or second to differentiation of the germline causes the individual to develop germline or somatic mosaicism. In family cases, the heterozygous 22q11.2 deletion has a 50% risk of recurrence (Fung et al., 2015). The incidence rate of deletion among the offspring of mosaic parents is uncertain, and it theoretically depends on the percentage of deleted gonadal cells (McMahon et al., 2015). Clinical characteristics of mosaic 22q11.2DS present from almost normal to serious. Asymptomatic mosaic 22q11.2DS is usually unnoticeable and contributes to the occurrence of an inherited deletion (Halder, Jain, Kabra, & Gupta, 2008). Through literature consulting, only five cases of a mosaic 22q11.2 deletion affecting offspring have been reported. The first case of germline mosaicism of the 22q11.2 deletion with two of three offspring inherited the deleted form of chromosome 22 was reported in 1998 (Hatchwell, Long, Wilde, Crolla, & Temple, 1998). In the same year, Kasprzak reported that a germline and somatic mosaic female (lymphocytes fluorescence in situ hybridization [FISH]: 7% deleted) had two sons with the 22q11.2 deletion (Kasprzak et al., 1998). In another study, a female with a probable germline mosaic 22q11.2 deletion transmitted the deleted hemizygous to two of her three children (Sandrin‐Garcia et al., 2002). These studies demonstrated that germline mosaic parents have a high risk of affecting their offspring. It is worth mentioning that it was reported that a somatic mosaic 22q11.2 deletion father (lymphocytes FISH: 19% deleted) had a mosaic son (lymphocytes FISH: 43% deleted) who had cardiac malformations (Chen et al., 2004). Another case of a mosaic mother (interphase and metaphase FISH: 10% deleted) and her mosaic fetus (heart tissue FISH: 85% deleted) was reported, and the author hypothesized that the chromosome 22 inherited from mosaic deletion parents could be more susceptible to deletion (Patel, Gawde, & Khatkhatay, 2006).

In this study, we reported the first case, in China, of a somatic and suspected gonadal mosaic 22q11.2 deletion in a mother who caused recurrent fetal conotruncal defects in a family; one fetus had pulmonary atresia (PA), and another had TOF. This case has implications for genetic counseling for families with 22q11.2DS.

## 2 MATERIALS AND METHODS

### 2.1 Ethical compliance

This research was conducted in accordance with the ethical guidelines of the Declaration of Helsinki and approved by the Ethics Committee of the Children's Hospital of Fudan University, Shanghai Medical College of Fudan University before the commencement of the study. Informed consent was obtained from the participants who were involved in this research.

### 2.2 Cases and tissue collection

Clinical data such as pregnancy history of the case, ultrasound results of the fetus and an amniotic fluid sample of the fetus with TOF were collected from the International Peace Maternity and Child Health Hospital, Shanghai, China. Blood samples for the FISH study were obtained from the family at the Children's Hospital of Fudan University in Shanghai, China.

### 2.3 Chromosomal microarray analysis

Since an ultrasound examination revealed that the second fetus had structural abnormalities, a chromosomal microarray analysis (CMA) was performed to screen for abnormal chromosomal disease. Genomic DNA was extracted from the amniotic fluid sample. Then, the DNA was amplified, labeled, and hybridized using the CytoScan™ HD system (Affymetrix) following the manufacturer's protocol. The hybridization data were visualized and analyzed by the Affymetrix Chromosome Analysis Suite software package with a calling threshold at 20 consecutive probes encompassing 25 kb or more in length.

### 2.4 FISH

To trace the origin of the 22q11.2 microdeletion in the fetus, an interphase FISH was conducted on the family members. One milliliter of the peripheral blood was collected from the members of this family. The blood samples were washed three times with PBS, and 1ml hypotonic solution (75 mmol/l KCl) was added to the tube and incubated for 30 min at 37°C before fixation by a 3:1 methanol and acetic acid solution. Then, the cell suspensions were fixed on slides. A Vysis TUPLE1/ARSA kit (Vysis) was used according to the manufacturer's instructions (Lundin et al., 2010). The TUPLE1 probe that this kit contained is located in the 22q11.2 region overlapping the HIRA gene (OMIM 600237), and the ARSA probe is the control probe for the 22q13.3 region. The slides that were previously prepared were dehydrated and co‐denatured with probes at 73°C for 5 min. Then, hybridization was performed overnight at 37°C The slides were counterstained with 4',6‐diamidino‐2‐phenylindole (DAPI) (Vysis). Image analysis was performed using a CCD camera (ProgRes). Finally, 200 interphase nuclei were scored. The metaphase lymphocytes were obtained by blood culture using the standard cytogenetic techniques. The FISH practice is done as described in the methods above.

### 2.5 Clinical examination related to 22q11.2DS

To detect whether the mosaic 22q11.2DS patient had hidden cardiovascular abnormalities, immunodeficiency or endocrine abnormalities, an echocardiogram was conducted, and the parathyroid function, flow cytometric enumeration of the lymphocyte subsets and serum concentrations of immunoglobulin were measured.

## 3 RESULTS

### 3.1 Case description

**Figure 1:**
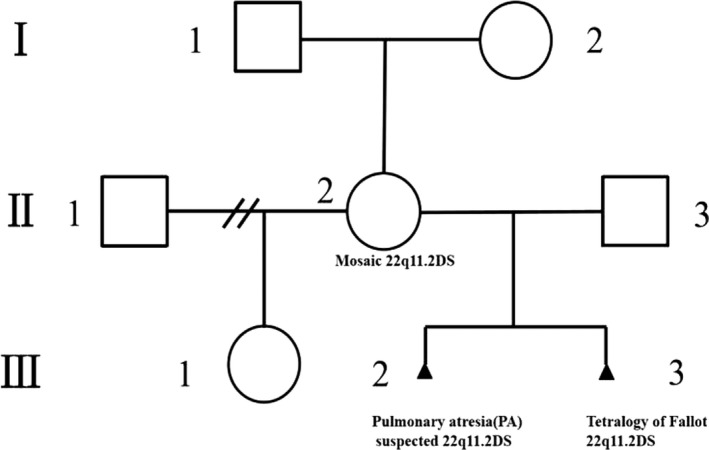
A pedigree of the Chinese family. The second fetus (III 3) was confirmed to have Tetralogy of Fallot (TOF), right renal agenesis, and 22q11.2DS. His sibling (III 2) had pulmonary atresia (PA). The mother (II 2) with no phenotype of cardiac defects had mosaic 22q11.2DS, as confirmed by fluorescence in situ hybridization

**Figure 2:**
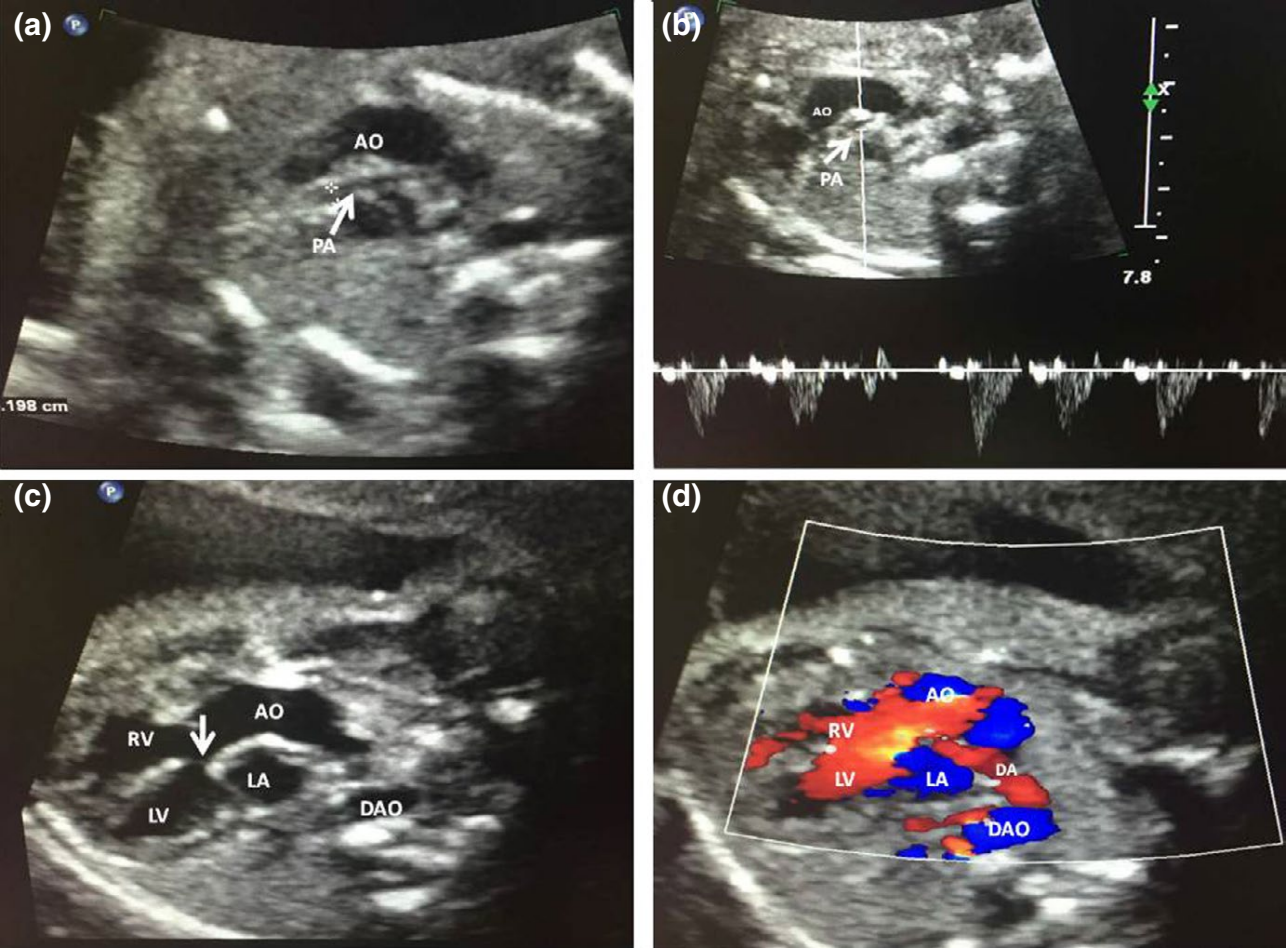
Echocardiographic images of the second fetus (Figure 1. III 3) demonstrate pulmonary artery stenosis (a, b) and aortic overriding and ventricular septal defects (c, d). AO, aorta; DA, ductus arteriosus; DAO, descending aorta; LA, left atrium; LV, left ventricle; PA, pulmonary artery; RV, right ventricle

**Figure 3:**
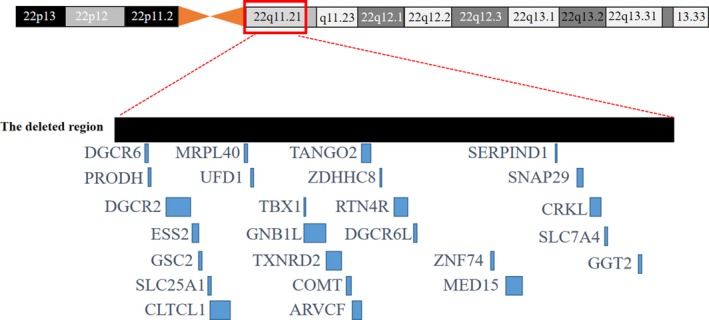
Gene alignment: deleted segments (chr22: 18636749– 21,800,471) of the second fetus (III 3) based on the UCSC Genome Browser custom track tools (hg19)

A 35‐year‐old woman (G3P1A2) was referred to the children's hospital of Fudan University, China for genetic counseling. She showed a normal phenotype and had a daughter with her former husband (Figure1 III1), who was in primary school and had no mental or behavioral disorders. After the woman was married to her present husband, she had two continuous fetuses with conotruncal defects: an ultrasound examination of the previous fetus (Figure1 III2) revealed PA (The general diagnosis was from the medical records which did not mention that whether the PA was with or without intact ventricular septum and ultrasound images were not obtained); the echocardiographic images of the other fetus (Figure1 III3) showed TOF (Figure2) and right renal agenesis that was suggestive of multiple malformations. Due to these recurrent conotruncal defects in the continuous fetuses, amniocentesis and a CMA on the fetus with multiple malformations were performed and revealed a microdeletion on 22q11.2 (Chr22:18636749‐ 21800471; Figure 3). Both pregnancies were terminated by the induction of labor at approximately 28 weeks of gestation at the couple's will. No detailed clinical records of the fetuses were available. The woman and her present husband were healthy and nonconsanguineous and had no family history of congenital defects. A pregnancy history of recurrent conotruncal defects and the CMA results alluded to familial 22q11.2DS. For further genetic counseling, we advised the couple and their parents to screen for the 22q11.2 microdeletion.

### 3.2 The 22q11.2 microdeletion screen in the family members

**Figure 4:**
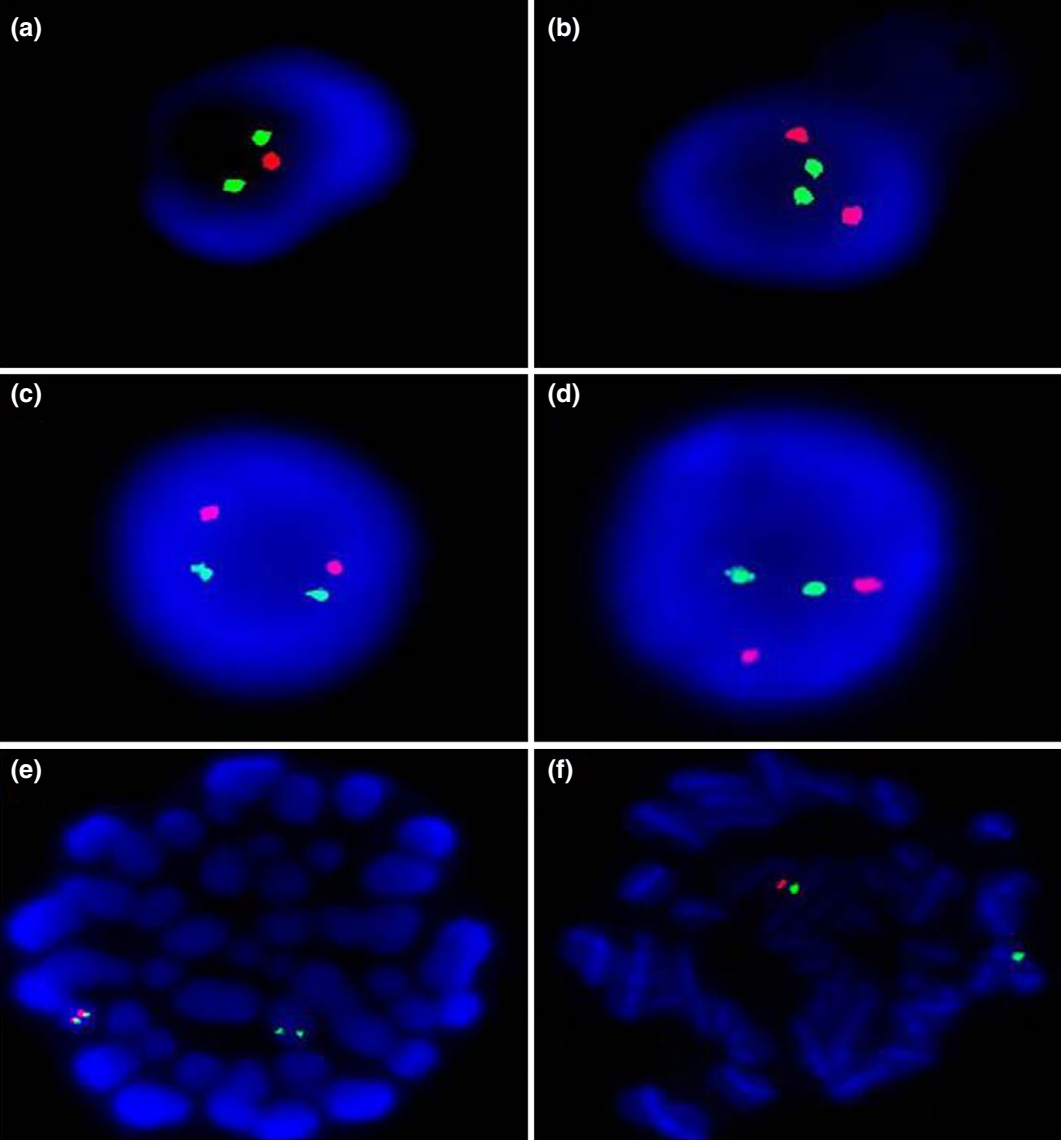
Fluorescence in situ hybridization (FISH) analysis of the interphase nucleated blood cells shows a deletion on 22q11.2 in the fetuses’ mother (a) and normal signals in the fetuses’ father (b) and fetuses’ grandparents (c, d); FISH analysis of the metaphase lymphocytes: hemizygous deletion signals were observed in more than 10% of the metaphase lymphocytes (e, f). Red signal: 22q11.2; green signal: 22q13

To trace the origin of the 22q11.2 microdeletion of the fetus, interphase FISH was performed previously on his parents. The results of the mother showed that 164 (82%) of the nucleated blood cell nuclei had normal signals and that 36 (18%) of the nuclei had a hemizygous deletion on the 22q11.2 locus (Figure 4a). The FISH analysis of the fetuses’ father displayed normal signals in approximately 95%‐97% of the nuclei (Figure 4b). To detect whether the mosaic deletion of the mother was de novo or inherited, a FISH was performed on the grandparents. The results revealed that they carried no deletion (Figure4c,d), which suggested that the deletion occurred de novo.

### 3.3 Mosaicism defined in the metaphase cells

To confirm the 22q11.2DS mosaicism of the mother, metaphase FISH was performed and hemizygous deletion signals were shown in more than 10% of the metaphase cells (Figure 4). This result supported the diagnosis of 22q11.2DS mosaicism in the mother. Thus, as the female has a risk of the 22q11.2 microdeletion in her next pregnancy, we may advise her to choose natural conception under 22q11.2 microdeletion screening early in her pregnancy, or to use in vitro fertilization.

### 3.4 Clinical characteristics of the mosaic 22q11.2DS mother

The mother is of a relatively short stature (1.5 m) and has no obvious facial abnormalities. She has no behavioral or mental problems. She does not have a history of frequent respiratory infections or seizures. The endocrine studies showed that she has hypocalcemia (2.21 mmol/l) and hypophosphatemia (0.99 mmol/l) but normal parathyroid hormone levels (5.2 pmol/l). Immunological testing revealed a moderately low percentage of CD4 + T helper cells (percentage: 23.86%, absolute counts: 404.36 μl). Quantitative immunoglobulin studies were done and showed normal results. The complete test results are shown in Table 1. These examination results showed that the mother presented mild abnormalities but no clinical problems.

**Table 1:** The results of clinical testing of the proband's mother

Tests Items	Results	Normal range^a^
Calcaemia	2.11 mmol/L	2.2–2.65 mmol/L
Phosphorus	0.99 mmol/L	1.29–2.26 mmol/L
Parathyroid hormone	5.2 pmol/L	1.06–7.31 pmol/L
CD3 + T cell	Percentage: 65.42%	56.09%–84.32%
	Absolute counts: 1,108.8	723–2271
CD 4 + T cell	Percentage: 23.86%	28.06−53.59%
	Absolute counts: 404.36	396–1309
CD 8 + T cell	Percentage: 27.27%	16.37%–42.65%
	Absolute counts: 462.21	224–1024
CD19 + B cell	Percentage: 10.62%	7.19−25.85%
	Absolute counts: 179.3	118–645
CD56 + NK cell	Percentage: 23.37%	3.66%–26.74%
	Absolute counts: 396.09	61–607
CD4/CD8 Ratio	0.87	0.71–2.82
IgA	2.78 g/L	0.7–3.5 g/L
IgG	15.6 g/L	7.0–16.6 g/L
IgM	1.4 g/L	0.5–2.6 g/L

a The reference range for the lymphocyte subsets followed the Reference ranges for lymphocyte subsets among healthy Hong Kong Chinese adults by single‐platform flow cytometry. (Wong et al., 2013).

## 4 DISCUSSION

In the present study, a CMA of the fetus with TOF revealed a microdeletion on the 22q11.2 locus. No more genetic evidence was available for the fetus with PA, but his conotruncal defects phenotype may have occurred from 22q11.2DS. Further studies have been carried out to investigate whether or not the deletion was inherited. FISH was performed on this family and showed that the asymptomatic mother had somatic mosaicism of the 22q11.2 microdeletion, which was de novo. The presence of the deleted form of the chromosome 22 hemizygote in the offspring confirmed that the germ cells of the female were involved in the 22q11.2 microdeletion, which suggests that she had a somatic and suspected gonadal mosaicism. Taken together, we reported that a mosaic 22q11.2 microdeletion in a Chinese family caused recurrent fetal conotruncal defects: first fetus with PA and a second fetus with TOF and right renal agenesis.

FISH analysis is a precise method to detect mosaicism of the 22q11.2 microdeletion, especially for a low‐level mosaicism (<35%; Chen et al., 2017). In our study, interphase and metaphase FISH were performed on nucleated blood cells to confirm the diagnosis of low‐level mosaicism in the mother. Her pregnancy history of continuous fetuses with conotruncal defects suggested that her gonadal cells were influenced. Mosaicism in common microdeletion syndromes has usually been reported as case reports (Huynh et al., 2017). Conversely, a study in India showed that mosaicism was not rare in 22q11.2DS (28.2%; Halder et al., 2018). Mosaicism has been shown in the literature to range from fatal to asymptomatic (Halder et al., 2008). Thus, this not low incidence of mosaicism in 22q11.2DS, especially those with no or slight clinical features, may contribute to the recurrence of 22q11.2DS. For familiar 22q11.2DS, the risk of recurrence of the heterozygous 22q11.2 deletion was under a 50% (Fung et al., 2015). However, the recurrence rate in the offspring of mosaicism was uncertain. In this case, two of three offspring inherited the deletion heterozygotes from the mosaicism. Moreover, the mother had almost none of the clinical characteristics that are associated with 22q11.2DS except for some insignificant abnormal results in her laboratory examinations. This finding suggested that the load of deletion cells in the gonads may be higher than that of the blood. Consistent with our findings, in other cases of mosaic parents influencing their offspring, all mosaic parents have a low‐level (<35%) of mosaicism in their blood cells (Kasprzak et al., 1998; Patel et al., 2006; Sandrin‐Garcia et al., 2002). Hence, parents with low levels of mosaicism in blood cells may be at risk of influencing their children.

The phenotypic spectrum of 22q11.2DS was wide and ranges from asymptomatic to fatal. In the present report, the mosaic mother had none of the typical symptoms of 22q11.2DS such as congenital anomalies, which explains her late diagnosis. For the same reason, many asymptomatic patients have been diagnosed after the birth of an affected offspring (Fung et al., 2015). However, the extended laboratory examinations on this mother showed some moderate abnormalities, including hypocalcemia, hypophosphatemia, and low percentage of CD4 + T helper cells. A study of adults with 22q11.2DS reported that 80% of patients had a history of hypocalcemia (Cheung et al., 2014) and attributes of hypoparathyroidism. A low immune cell count is also common among patients with 22q11.2DS (McDonald‐McGinn & Sullivan, 2011). Furthermore, the first fetus was diagnosed with PA, and the second fetus with the confirmed 22q11.2DS had TOF and right renal agenesis. These two conotruncal defects have frequently been reported in patients with 22q11.2DS. A study from 1992 to 2018 of a cohort of 1,421 patients with confirmed 22q11.2DS showed that 11.52% of patients had TOF, and 16% had renal anomalies (Campbell et al., 2018). *TBX1* (OMIM 602054), *CRKL* (OMIM 602007), and *MAPK1* (OMIM 176948) haploinsufficiency on the 22q11.2 locus have been reported to be highly associated with the syndrome (Koczkowska et al., 2017). A recent study identified a recurrent 370‐kb deletion that includes CRKL (OMIM 602007) on the 22q11.2 locus to be critical to the renal abnormality phenotype (Lopez‐Rivera et al., 2017).

Our study has several limitations. First, because no additional clinical data were obtained and no genetic studies were performed for the first fetus, we could not describe any similarities or dissimilarities between the fetuses with 22q11.2DS. Second, since either a polar body or oocyte would be virtually impossible to obtain, the gonadal mosaicism in the mother was not assessed. Third, the family's first girl (Fig1. Ⅲ1) did not undergo clinical examination and genetic testing for 22q11.2DS because of her mother's refusal, so we could not get clinical information and genetic test results about the this girl.

In conclusion, this was the first study to report the case of a somatic and suspected gonadal mosaic 22q11.2 microdeletion in a female affecting her two fetuses those with conotruncal defects in China. It demonstrates that, in a family with suspected 22q11.2DS offspring, especially conotruncal defects, asymptomatic parents have the risk of carrying a mosaicism. The interphase FISH is the primary method to screen for mosaicism. Furthermore, this study suggests that a history of hypocalcemia and low immune cell counts are important clinical features of mosaic 22q11.2DS. As 22q11.2DS refers to multiple malformations, we stress that genetic counseling and associated examinations should be offered to mosaic parents, even those who show no phenotype.

## CONFLICT OF INTERESTS

The authors declare that they have no competing interests.
